# High-risk human papillomavirus genotype distribution among women with gynecology complaints in northwest Ethiopia

**DOI:** 10.1186/s13027-023-00481-3

**Published:** 2023-01-27

**Authors:** Awoke Derbie, Melanie Maier, Bereket Amare, Eyaya Misgan, Endalkachew Nibret, Uwe G. Liebert, Yimtubezinash Woldeamanuel, Tamrat Abebe

**Affiliations:** 1 Department of Medical Microbiology, College of Medicine and Health Sciences, Bahir Dar University, Bahir Dar, Ethiopia; 2 Centre for Innovative Drug Development and Therapeutic Trials for Africa (CDT-Africa), College of Health Sciences, Addis Ababa University, Addis Ababa, Ethiopia; 3 Department of Health Biotechnology, Institute of Biotechnology, Bahir Dar University, Bahir Dar, Ethiopia; 4 Department of Medical Microbiology, Immunology and Parasitology, School of Medicine, College of Health Sciences, Addis Ababa University, Addis Ababa, Ethiopia; 5 Department of Diagnostics, Institute of Virology, Leipzig University Hospital, Leipzig, Germany; 6 Department of Pathology, College of Medicine and Health Sciences, Bahir Dar University, Bahir Dar, Ethiopia; 7 Department of Gynecology and Obstetrics, College of Medicine and Health Sciences, Bahir Dar University, Bahir Dar, Ethiopia; 8 Department of Biology, College of Science, Bahir Dar University, Bahir Dar, Ethiopia

**Keywords:** Human papillomavirus (HPV), HR-HPV, Genotype distribution, Northwest Ethiopia

## Abstract

**Background:**

Human papillomavirus (HPV) genotypes differ by geographic location. With the advent of HPV vaccination and HPV-based cervical screening tests in Ethiopia, a nationwide dataset on the genotype distribution of HPV among women has paramount importance in the fight against cervical cancer. However, there is limited data in this regard in the northwest part of the country. Therefore, this study aimed to identify the genotype distribution of high-risk HPVs among women presenting with cervical abnormalities.

**Methods:**

A health facility-based cross-sectional study was conducted at Felege Hiwot Comprehensive Specialized Hospital (FHCSH), Bahir Dar–Ethiopia. Women aged ≥ 30 years who visited the hospital gynecology unit from 01 March 2019 to 30 October 2021 were included. Following general and pelvic examinations, a senior gynecologist collected cervical punch biopsies for histopathological examinations and cervical swabs for HR-HPV detection using the Abbott Alinity m system (Abbott Molecular, Des Plaines, IL, USA). Extended genotyping was carried out with the INNO-LiPA HPV Genotyping Extra II assay (INNO-LiPA; Fujirebio Europe, Ghent, Belgium) as per the manufacturer protocols at the Institute of Virology, Leipzig University Hospital, Germany.

**Results:**

We included 355 women with a mean age of 46.4 ± 11.4 years. The majority of the participants, 277 (79.4%) were sexually active before the age of 18 years and 180 (51.6%) had multiple sexual partners. Forty-eight (13.5%) of the participants were HIV positive. The proportion of HR-HPV was 53.0% (n = 188; 95%CI: 47.8–58.1%). From these samples, 13 different HR-HPV types with a total of 258 sequences were identified. The detection of HR-HPV increased significantly with an increase in the age of the participants. The predominant identified HR-HPV was HPV16, 50.4% followed by HPV31 (9.7%), HPV33 (8.5%), HPV39, and HPV68 each (5.8%) and HPV18 (4.7%). Of the total HR-HPV-positive women, 23.9% (45/188) were infected with multiple HR-HPV types. All HPV16, HPV18, HPV35, and HPV45 genotypes (as a single or in coinfections) were found to be associated with either high-grade lesions or cervical cancer.

**Conclusions:**

HR-HPV infection was reportedly higher among women in the present study area. Based on our findings, we strongly recommend the nonavalent HPV vaccine for immunization and any HPV-based screening method to take into consideration the predominant genotypes circulating in the country. The role of multiple HPV infections in high-grade cervical lesions entails further study in Ethiopia.

## Background

Cervical cancer is one of the most frequent malignancies worldwide. Almost nine out of ten deaths occur in developing countries, where there is inefficient vaccination and cervical screening [1]. Cervical cancer is one of the emerging public health challenges in Ethiopia. The incidence and prevalence are increasing [2]. According to the international agency for research on cancer assessments, the estimated number of new cervical cancer cases at 7500 in 2020 could intensify to 15,300 in 2040. Similarly, the mortality from the disease could increase from 5340 in 2020 to 11,000 in 2040 yearly in Ethiopia [3].

Epidemiological, clinical, and molecular-based studies confirmed that persistent infection with oncogenic types or high-risk (HR) human papillomaviruses (HPVs) is the main cause of cervical cancer [4, 5]. HR-HPVs are also linked with a large number of other types of cancers (anus, vulva, vagina, and penis) and a growing number of head and neck tumors [6–10]. So far, more than 200 different HPVs are characterized and completely sequenced. Of all types, about 40 are sexually transmitted [8, 11–14]. HPV16, 18, 31, 33, 35, 39, 45, 51, 52, 56, 58, 59 and 68 grouped under HR-HPV because of their strong association in carcinogenesis. Infection with HPV type 16 or HPV18 is associated with a higher risk of disease progression compared to other HR-HPVs causing more than 50%  of all cervical cancer burdens globally [15–18]. According to a report from a recent systematic review, HPV16 followed by HPV52, HPV35, HPV18, and HPV56 were the most common types identified from cervical samples in Ethiopia. The combined prevalence of HPV 16/18 was 53.7% [19].

Cervical cancer is preventable. Early detection of precancerous lesions and vaccinating schoolchildren with optimal coverage could save lives. HPV-based screening is rarely available in Ethiopia. Vaccination was introduced in 2018 using Gardasil-4® recombinant vaccine that targets HPV 6, 11, 16, and HPV18 for 14-year-old girls and according to the 2020 estimation, the first and last dose coverage was 76% and 95%, respectively [1, 20]. Despite its proven effectiveness and safety, the currently available vaccine in Ethiopia presents some limitations in terms of incorporating the most important oncogenic HPV variants circulating in the country.

HPV types differ by geographical location and the host’s background [21]. The burden and heterogeneity in HPV distribution between and among countries and even among specific regions within a country are significantly different [22], which directly influence the HPV-based screening and vaccination approaches. With the advent of HPV HPV-based screening tests and vaccination in Ethiopia, it is crucial to map the distribution of HPV genotypes in areas where there is no previous report. Even though there are few studies conducted in Ethiopia on HPV genotyping [23–31], to the best of our knowledge, there are only a couple of health facility-based reports in the northern part of the country [24, 31].

Therefore, this study was conducted to identify the HR-HPV genotype circulating in northwest Ethiopia among women presenting with various cervical lesions. The finding will be an important input to help guide in the revision of the current national cervical screening and vaccination program as national data and context is important in planning full-pledged cervical cancer prevention services.

## Methods and materials

### Study setting

A hospital-based cross-sectional study was conducted at Felege Hiwot Comprehensive Specialized Hospital (FHCSH) between 01 March 2019 and 30 Oct 2021. The hospital is located in Bahir Dar city, northwest Ethiopia, which is the capital of Amhara National Regional State, positioned about 565 km away from Addis Ababa. The FHCSH, with more than 500 beds, is a tertiary health care facility that provides several types of specialized referral services for more than ten million people of northwest Ethiopia.

### Study population

Cervical specimens were collected from 355 women presented with suggestive signs and symptoms of abnormal cervical lesions including abnormal vaginal discharge, vaginal bleeding, and women complaining of painful sexual intercourse [32, 33]. Participants with the following characteristics were considered to take part in the study: Age ≥ 30, had sexual history, were not pregnant, have an intact uterus and cervix, and not on menses. On the other hand, women who were seriously ill and those who were under treatment for invasive cervical cancer were excluded.

### Data and sample collection

An interviewer-administered structured questionnaire was used to collect data on the participants’ demographic and gynecologic history. The tool was prepared following previous similar works [6, 34–39]. Trained nurses who were working in the hospital gynecology department collected the questionnaire-based data. Each participant underwent a general and pelvic examination in a compassionate and respectful process after obtaining an informed consent. Then, from the grossly visible lesion, a licensed gynecologist collected a punch biopsy for histopathological examinations and cervical swabs with a single-use broom-type brush (Digene HC2 DNA collection device: Qiagen, Hilden, Germany) following the manufacturer’s instruction for HPV DNA testing. Specimen Transport Medium (STM) that contains 0.05% sodium azide as a preservative accompanied the sample collection brush. Cervical swabs were labeled with a unique code and the patient card number and were stored at − 80 °C at Bahir Dar University, College of Medicine and Health Science Research Laboratory. Later, the frozen specimens were transported on dry ice packs to the Institute of Virology, Leipzig University Hospital, Germany for molecular analysis.

### HPV DNA detection and genotyping

The molecular detection and characterization of HPVs from cervical lesions were based on the principle that the viral DNA is present in the epithelial layers of the affected tissue and can be detected easily with PCR-based technologies [40]. HR-HPV detection and characterization were made using Alinity m HR HPV AMP Kit on Alinity m System (software version: 1.6.3) (Abbott Molecular, Des Plaines, IL, USA). The Alinity m System offers fully automated continuous random access to different assays. It is a fully integrated and automated molecular analyzer introduced in 2019 [41]. Alinity m HR-HPV assay fulfills the international consensus guideline criteria for primary cervical cancer screening [42]. The time to the first result is less than two hours [43]. Using real-time PCR and ReadiFlex® technology, the Alinity m HR HPV assay is a qualitative in vitro test targeting the conserved L1 region for the detection of DNA from 14 HR-PV genotypes; HPV16, 18, 31, 33, 35, 39, 45, 51, 52, 56, 58, 59, 66, and 68 from clinical specimen [42]. HR-HPVs were detected with genotype-specific probes in five distinct channels: HPV16, HPV18, HPV45, and other high-risk genotypes group A (HPV31, 33, 52, and 58), and other high-risk genotypes group B (HPV35, 39, 51, 56, 59, 66, and 68) at clinically relevant infection levels [41]. To ensure its quality performance, the Alinity m system in addition to testing external positive and negative controls, detects the endogenous human beta-globin sequence as a Cellular Control signal to evaluate cell adequacy, sample extraction, and amplification efficiency [43].

The steps of the Alinity m HR HPV assay consist of sample preparation, real-time PCR assembly, amplification/detection, and result calculation and reporting. All steps of the assay are performed automatically by the Alinity m system. During sample preparation, 400 µL of cervical swabs were pretreated and lysed with chaotropic reagents, allowing HPD DNA to be captured on magnetic microparticles. The bound purified DNA was then washed and eluted. A lyophilized amplification master mix consisting of DNA polymerase, primers, probes, and dNTPs rehydrated using the eluate and activation reagent. The resultant PCR mixture was then transferred to a reaction vessel, which was subsequently capped and transferred to the amplification and detection unit. Results are reported automatically [44, 45].

Samples (n = 49) that tested positive for HR-HPV A and HR-HPV B with the Abbott Alinity m test were further processed using the INNO-LiPA HPV Genotyping Extra II assay (INNO-LiPA; Fujirebio Europe, Ghent, Belgium) for identification of the specific genotypes following the manufacturer instructions and as described previously [46, 47].

With regard to the histological examination, after collection, cervical biopsies were placed in screw-capped and labeled bottles that contained 20 ml of 10% formol-saline fixative solution and transported to the hospital pathology laboratory for downstream processing by the senior pathologist as described previously [48]. Lastly, the microscopic examination of slides was reported as normal histology, cervicitis, Cervical intraepithelial neoplasia (CIN)1, CIN2, CIN3, cancer, and other miscellaneous findings.

### Data analysis

Generated data were entered and analyzed using SPSS V25. Descriptive statistics were used to describe the demographic and clinical characteristics of the study participants. The type distribution of the identified HR-HPVs with a 95% confidence interval (CI) was calculated. The results are presented as simple counts, percentages, and mean with standard deviations.

## Results

### Demographic and other characteristics of the study participants

In this study, 355 women were included. At enrolment, the study participants were in the age range of 30–80 years with a mean age of 46.4 ± 11.4 years. The majority of the participants were married 272 (76.6%), housewives 314 (88.5%), were from rural settings 232 (65.9%), and did not have formal education 272 (76.6%) (Table 1).

**Table 1:** Distribution of the study participants by their demographic and clinical characteristics, Bahir Dar, 2021

Characteristics	n (%)
Age groups (in years)	
Mean, SD	46.4, 11.4
30–40	142 (40.0)
41–50	115 (32.4)
> 50	98 (27.6)
Permanent residence	
Urban	121 (34.1)
Rural	232 (65.9)
Marital status	
Single	4 (1.1)
Married	272 (76.6)
Divorced	38 (10.7)
Other	40 (11.3)
Educational status	
No formal education	272 (76.6)
Primary	46 (13.0)
Secondary	17 (4.8)
Tertiary	20 (5.6)
Type of occupation	
House-wife	314 (88.5)
Private employee	17 (4.8)
Government employee	22 (6.2)
Other (prostitute)	2 (0.6)
Age at first sexual intercourse	
Mean, SD	15.7, 2.6
< 18 years	277 (79.4)
≥ 18 years	72 (20.6)
Life-time number of sexual partners	
Mean	1.7
1	169 (48.2)
≥ 2	180 (51.6)
HIV sero-status	
Positive	48 (13.5)
Negative	301(84.8)
Unknown	6 (1.7)
Previously treated for vaginal discharge	
Yes	106 (30.0)
No	247 (70.0)
Aware of cervical cancer	
Yes	144 (40.6)
No	211 (59.4)
How is the pathogen that causes cervical cancer transmitted?	
Sexually	11 (8.9)
Do not know	114 (91.1)
History of cervical screening	
Yes	97 (27.3)
No	258 (72.7)
History of cervical screening in the last five years	
Yes	88 (24.8)
No	267 (75.2)
HR-HPV	
Detected	188 (53.0)
Not detected	167 (47.0)

The majority of the participants 277 (79.4%) were sexually active before the age of 18 years. The mean age at first sexual intercourse was 15.7 ± 2.6. Furthermore, 48 (13.5%), 106 (30.0%), and 180 (51.6%) of the participants were HIV positive, had a history of vaginal discharge, and multiple sexual partners, respectively.

More than half of the participants 211 (59.4) were unaware of cervical cancer. The participants’ cervical screening practice and screening in the last five years were 97 (27.3%) and 88 (24.8%), respectively. Besides, their knowledge about the disease was very limited and only 11 (8.9%) of them know that the disease is caused by a sexually transmitted pathogen (Table 1).

### Type of the identified HR-HPVs

The prevalence of high-risk HPV (HR-HPV) was 53.0% (188/355; 95% CI: 47.8–58.1%). From the processed samples, 13 different HR-HPVs with a total of 258 sequences were identified. The detection of HR-HPV increased significantly with an increase in the age of participants. Similarly, HR-HPV detection was different based on the educational status and screening history of the study participants (*p*-value < 0.05) (Table 2).

**Table 2:** The detection rate of HR-HPV genotypes across socio-demographic, sexual behavior, and clinical variables, Bahir Dar, 2021

Variables	HR-HPV		*P-*value
	Detected, n (%)	Not detected, n (%)	
Age group			
30–40	57 (16.1)	85 (23.9)	< 0.001
41–50	64 (18.0)	51 (14.4)	
> 50	67 (18.9)	31 (8.7)	
Marital status			
Single	4 (1.1)	0	0.01
Married	131 (36.9)	141 (39.7)	
Divorced	25 (7.0)	13 (3.7)	
Other	27 (7.6)	13 (3.7)	
Educational status			
No formal education	147 (41.4)	125 (35.2)	0.01
Primary	17 (4.8)	29 (8.2)	
Secondary	14 (3.9)	3 (0.8)	
Tertiary	10 (2.8)	10 (2.8)	
Age at first sexual practice			
< 18	150 (43)	127 (36.4)	0.53
> 18	36 (10.3)	36 (10.3)	
Lifetime number of sexual partner			
1	88 (25.2)	81 (23.2)	0.73
> 2	97 (27.8)	83 (23.8)	
HIV sero-status			
Positive	26 (7.3)	22 (6.2)	0.78
Negative	158 (44.5)	142 (40)	
Unknown	4 (1.1)	2 (0.6)	
Cervical screening history			
Yes	42 (11.8)	55 (15.5)	0.02
No	146 (41.1)	112 (31.5)	
Screening in the last five years			
Yes	37 (10.4)	51 (14.4)	0.01
No	151 (42.5)	116 (32.7)	
Treated for vaginal discharge			
Yes	54 (15.2)	54 (15.2)	0.52
No	133 (37.5)	114 (32.1)	

**Fig. 1:**
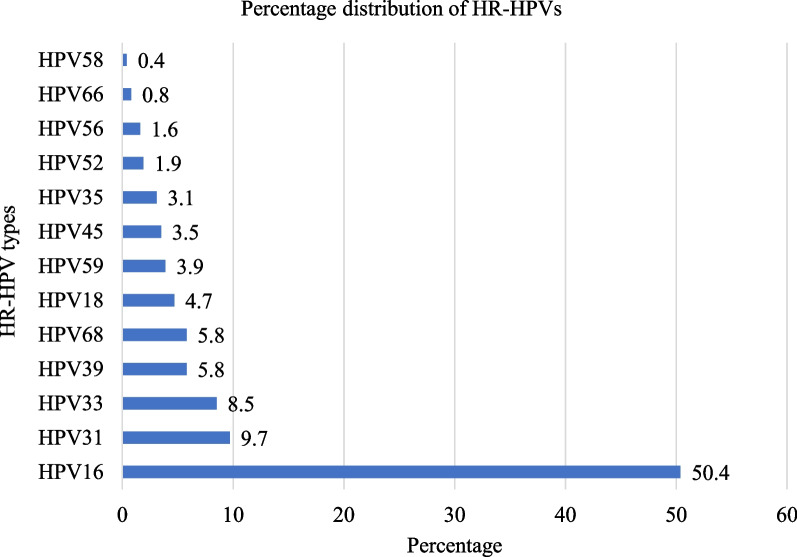
HR-HPV genotype distribution among women aged 30–80 years, northwest Ethiopia, 2021

The predominant identified HR-HPV was HPV16 that accounted for 50.4% (130/258: 95% CI: 29.4–39.2%) followed by HPV31 9.7% (25/258: 95% CI: 6.7–13.9%), HPV33 8.5% (22/258: 95% CI: 5.7–12.6%), HPV39 and HPV68 each 5.8% (15/258: 95% CI: 3.6–9.4%) and HPV18 4.7% (12/258: 95% CI: 2.7–8.0%). The least detected genotype was HPV58 (0.4%). The combined prevalence of HPV 16 and HPV18 was 55.1% (142/258: 95% CI: 48.9–61.0%) (Fig. 1).

### HR-HPV co-infections

Among HR-HPV positive samples (n = 188), mono-infection was documented in 143 (76.1%) whereas 27 (14.4%) and 18 (9.6%) samples were positive for two and multiple (≥ 3) HR-HPV types, respectively. We noted up to five different HR-HPVs infecting a single patient. The most common HR-HPV coinfection was HPV31 with HPV33 followed by HPV39 with HPV59 and HPV68.

### The distribution of HR-HPV in cervical lesions of different histopathological grades

Concerning the types of HR-HPVs involved in different histopathological grades, all HPV16, HPV18, HPV35, and HPV45 genotypes (as a single or in coinfections) were found to be associated with either high-grade lesions (CIN2+) or cervical cancer. Likewise, 79 (80.6%), 7 (87.5%), 3 (75%), and 5 (100%) of HPV16, HPV18, HPV35, and HPV45 mono infections were identified in histopathologically confirmed cancer cases, respectively. Similarly, 11 (78.6%) HPV31&33 coinfections were recovered from cancer cases.

Specifically, the detection of rate of HPV16 was increased with an increase in the degree of cervical lesions in which the proportion was 12.5% (n = 5), 16.7% (n = 2), 41.7% (n = 5), and 85.3% (n = 58) in Cervical Intraepithelial Neoplasia (CIN) 1, CIN2, CIN3, and cancer cases, respectively. In 9.7% (13/133) of cervical cancer cases, HR-HPVs were not detected (Table 3).

**Table 3:** The distribution of HR-HPVs in different cervical histopathologic grades, northwest Ethiopia, 2021

HPV type (s)	Histopathological classes					Total
	Normal	CIN1	CIN2	CIN3	Cancer	
HPV16	7	5	2	5	79	98
HPV18	1	0	0	0	7	8
HPV45	0	0	0	0	5	5
HPV35	0	0	0	1	3	4
HPV16, 45	0	0	0	0	2	2
HPV16, 56	0	0	0	0	1	1
HPV31, 33	2	0	0	1	11	14
HPV39, 59	0	0	0	0	1	1
HPV39, 68	0	1	0	1	1	3
HPV56, 66	0	1	0	0	0	1
HPV16, 31, 33	0	0	0	0	2	2
HPV18, 31, 33	0	0	1	0	0	1
HPV18, 39, 59	0	0	0	1	0	1
HPV31, 52, 66	0	1	0	0	0	1
HPV35, 39, 68	0	1	0	0	1	2
HPV35, 56, 68	0	0	0	0	1	1
HPV39, 59, 68	0	1	0	0	3	4
HPV16, 31, 33, 52	0	0	0	1	0	1
HPV16, 39, 59, 68	0	0	0	0	2	2
HPV18, 31, 33, 52, 58	0	0	0	0	1	1
HPV31, 52, 39, 59, 68	0	1	0	0	0	1
HPV not detected	58	29	9	2	13	111
Total	68	40	12	12	133	265

## Discussion

A nationwide dataset on the prevalence and genotype distribution of HPV among Ethiopian women is crucial as it affects the current vaccination and HPV-based screening. This, however, is not adequately available despite the high burden of cervical cancer-related morbidity and mortality in the country. Vaccination and screening-led elimination of cervical cancer are highly dependent on optimizing HPV data of a particular country. Such information is decisive to appraise the lasting impact of HPV vaccines and HPV-based screening, and informing policymakers on the best alternative options for cervical and other HPV-associated cancer prevention and control activities [49].

In the present study, the prevalence of HPV among women who presented with cervical abnormalities was 53.0% (188/355). In this regard, previously conducted similar studies in Ethiopia revealed wide-ranging findings. The proportion of HR-HPV among women who visited the gynecology clinics and who were recruited from the general population in different parts of Ethiopia previously ranged from 16 to 100% [23, 26, 27, 30, 31, 50]. A study by Gebremeskel et al*.* [31] that specifically included women from the northern part of the country, reported the proportion of HR-HPVs among the identified HPVs to be 55.5%, which is almost the same as our finding. However, according to our recent systematic review, the proportion of HR-HPV among women with different kinds of cervical abnormalities was 77.5% in Ethiopia [19]. The HR-HPV prevalence reported in other African countries was also diverse; in Togo 53.3% [51], and Zimbabwe 96% [52]. Variation in the proportion of HR-HPV across different studies might be because of the difference in the methods of HPV detection, study population, degree of cervical lesions, sociodemographic and other related factors [23, 26, 27, 30, 31, 50]

In our study, the HR-HPV proportion was higher among women aged above fifty years and HIV-positive women compared to their counterparts (*p*-value < 0.005) (Table 2). This implies that these groups of women should get priority for public health interventions such as, screening by the use of HPV-based and other similar methods and a positive HPV result at this age might implies persistence for a number of reasons [53].

In the present study, the leading identified HR-HPV was HPV16 (50.4%). In other similar reported series in Ethiopia, other parts of Africa, and globally at large, HPV16 was the single most common genotype identified in cervical samples. The second, third, fourth, and the rest most commonly reported genotypes were usually different. Specifically, HR-HPV types from cervical samples in Ethiopia were reported to be diverse [23, 26, 27, 30, 31, 50]. This implies that HPV genotype distribution is diverse at different places and periods even within the same population [54]. Similarly, the distribution of HR-HPVs among women in other African countries was reported to heterogeneous [25, 49, 52, 55, 56]. For instance, HPV16 is not the first-ranked genotype from cervical samples in Kenya [53], Togo [51, 56], and Nigeria [22]. According to studies, not only HPV16 but also HPV18 is not that important in some African countries [51, 56]. However, systematic reviews in Africa, Asia, Latin America, and North America consistently reported that HPV16 is the most frequently identified genotype among women with various degrees of cervical lesions [57–60]. The genotype distribution of other HPVs is heavily dependent on the type of cervical lesions. Most often, HPV18, HPV31, 33, 52 and 56 are found as confecting genotypes [61].

Globally, HPV18 is the most frequently detected HR-HPV next to HPV16 from advanced cervical lesions including cancer [18]. However, it was rare in our study and we reported a similar result in a recently published systematic review [19]. The reason behind the low involvement of HPV18 in cancer and high-grade cervical lesions in Ethiopia requires further studies. Generally, the difference in the proportion and type of HR-HPVs among different studies in Ethiopia and other parts of the world might vary partly due to study-specific characteristics, heterogeneity in geographical location [62], age, life style and socio-economic status of the study participants and differences in study population, and most importantly methods used for HPV detection [49, 53].

The understanding that persistent infection with certain HR-HPVs is mainly a cause of cervical cancer has resulted in the development of new prospects for cervical screening and vaccination globally. HPV-based testing for cervical screening is becoming a cost-effective approach in most countries around the world [63]. In 2020, the World Health Organization (WHO) launched a global strategy to accelerate the elimination of cervical cancer as a public health problem. According to this strategy, priority is given to vaccinating schoolgirls, screening, and treatment of precancerous lesions [64]. This milestone approach is primarily dependent on the availability of nationwide HPV genotype data for countries, like Ethiopia. Our finding together with other previous HPV genotype reports could be used to partly evaluate the impact of vaccination and in the future the impact of the screening program in Ethiopia [62].

The current vaccine cocktail used in the country since 2018 for schoolgirls is Gardasil-4® that targets HPV6, 11, 16, and 18. The vaccine does not target other HR-HPVs circulating in the country. In the present study, for example, the combined prevalence of HPV16 and HPV18 was 55.1%, which implies that a significant proportion of girls might not be protected against other types even though they are vaccinated. So far, HPV vaccination is not part of the national immunization program in Ethiopia. One reason may be the cost of the vaccine. A recent study revealed that compared to the Gardasil-4™, a nonavalent Gardasil®9 that targets close to 90% of all HR-HPVs is a profitable option for Ethiopia [65].

A large proportion of HPV infections are sustained by multiple genotypes [66]. Of the total HR-HPV-positive women, 23.9% (45/188) were infected with two and more (up to five) multiple HR-HPVs in the present study. A study in China similarly reported 29.8% HR-HPV multiple infections from women with abnormal cervical cytology [67]. Multiple HPV infections are usually common at a younger age [66]. However, the role of such multiple infections in cervical carcinogenesis has not been well explained in Ethiopia. Wentzensen et al*.* reported up to 14 HPV types from a single cervical specimen although they did not observe type interactions among multiple HPVs [68]. In the present study, we noted multiple HR-HPV infections without the development of high-grade lesions. For instance, infections with HPV31, 52, 66, and infections with HPV31, 52, 39, 59, and 68 were not found to be associated with any form of high-grade cervical lesions. The role of multiple HR-HPV infections in the development of an advanced form of cervical lesions including the potential efficacy of HPV vaccines on such infections warrants further research in Ethiopia [30].

There are contradicting reports about the role of multiple HR-HPV infections in cervical carcinogenesis. Adcock and his colleague reported that HPV genotype and viral load, but not a multiplicity of HPV infections are important predictors of high-grade lesions including cervical cancer [69]. In contrast, a recently published study (2021) by Kim et al*.* showed that multiple HPV infections were found significantly associated with high-grade cervical lesions compared to infections with single HPV types. Furthermore, according to Kim et al. report, patients with multiple HPV infections exhibited a persistent and longer duration of the infection compared to patients with a single HPV infection [70].

Concerning the identified HR-HPVs and histopathologic grades, all HPV16, HPV18, HPV35, and HPV45 were associated with either high-grade lesions (CIN2+) or cervical cancer in the present study. Likewise, 79 (80.6%), 7 (87.5%), 3 (75%), and 5 (100%) of HPV16, HPV18, HPV35, and HPV45 were respectively noted in histopathologically confirmed cervical cancers. Specifically, the detection of HPV16 was increasing with the degree of cervical lesions in which the proportion was 5 (12.5%), 2 (16.7%), 5 (41.7%), and 58 (85.3%) in Cervical intraepithelial neoplasia (CIN)1, CIN2, CIN3, and cancer, respectively. A statistically significant association between HPV16 and the progression of cervical lesions was reported by a previous study [71]. Similarly, in our study 11 (78.6%) HPV31&33 coinfections were recovered from cancer cases. A study showed that next to HPV16, the hierarchy of HPV types based on their carcinogenic potential was reported to be HPV33 followed by HPV31 [69]. Song et al*.* in China also discovered that the prevalence of HPV16 and HPV33 increased significantly with the degree of cervical lesions [67].

Finally, according to the latest WHO report (2022), a large proportion of cervical cancer cases (> 95%) are due to infection with HPV [18]. In our study, 9.7% (13/133) of cervical cancer cases were without HR-HPVs. A study in Belgium showed that up to 15% of cervical cancers were reported to be without HPV infection [72]. Additional study is required in this regard in Ethiopia.

## Conclusions

This study provided a health facility-based estimate of HR-HPV infection of the uterine cervix and the common genotypes associated with the infection in northwest Ethiopia. More than half of the study participants tested positive for HR-HPV mainly infected with HPV16, HPV31 and HPV33. About 24% of cervical samples were found to be infected with two and more (up to five) multiple HR-HPV types. The HR-HPV proportion was higher among women aged above 50 years and HIV-positive once. Therefore, all forms of cervical cancer prevention strategies including multivalent HPV vaccination and screening should be expanded in the study area. Community-based similar studies with better HPV detection methods should be considered for a better appreciation of the HPVs circulating in northwest Ethiopia.

## Data Availability

The original data source could be shared upon the request of the principal investigator.
